# Renal effects of metallothionein induction by zinc *in vitro* and *in vivo*

**DOI:** 10.1186/s12882-017-0503-z

**Published:** 2017-03-16

**Authors:** Moritz Schanz, Lea Schaaf, Juergen Dippon, Dagmar Biegger, Peter Fritz, Mark Dominik Alscher, Martin Kimmel

**Affiliations:** 1 Department of Internal Medicine, Division of General Internal Medicine and Nephrology, Robert-Bosch Hospital Stuttgart, Auerbachstraße 110, 70376 Stuttgart, Germany; 2 Department of Mathematics, University of Stuttgart, Stuttgart, Germany; 3 Dr. Margarete Fischer-Bosch Institute of Clinical Pharmacology, Stuttgart, Germany

**Keywords:** Cell culture, Contrast medium, Hypoxia, Induction, Metallothionein, Zinc

## Abstract

**Background:**

Metallothionein (MTT) is an endogenous antioxidant that can be induced by both zinc (Zn) and ischemia. In kidneys, increased MTT expression exerts a putative protective role in diabetes and hypoxia. Our goal was to further investigate the behavior of MTT under the influence of Zn and hypoxia *in vitro* and *in vivo*.

**Methods:**

MTT expression was measured *in vitro* in cell cultures of proximal tubular cells (LCC-PK1) by immune-histochemistry and real-time PCR after incubation with increasing concentrations of Zn under hypoxic and non-hypoxic conditions. In addition, *in vivo* studies were carried out in 54 patients to study MTT induction through Zn. This is a sub-study of a prospective, randomized, double-blind trial on prevention of contrast-media-induced nephropathy using Placebo, Zn and N-Acetylcysteine. Blood samples were obtained before and after 2 days p.o. treatment with or without Zn (60 mg). ELISA-based MTT level measurements were done to evaluate the effects of Zn administration. For *in vivo* analysis, we considered the ratio of MTT to baseline MTT (MTT_1_/MTT_0_) and the ratio of eGFR (eGFR_1_/eGFR_0_), correspondingly.

**Results:**

*In vitro* quantitative immuno-histochemical analysis (IHC) and real-time PCR showed that at increasing levels of Zn (5, 10, and 15 μg/ml) led to a progressive increase of MTTs: Median (IQR) expression of IHC also increased progressively from 0.10 (0.09–0.12), 0.15 (0.12–0.18), 0.25 (0.25–0.27), 0.59 (0.48–0.70) (*p* < 0.0001). Median (IQR) expression of PCR: 0.59 (0.51–1.72), 1.62 (1.38–4.70), 3.58 (3.06–10.42) and 10.81 (9.24–31.47) (*p* < 0.0001). In contrast, hypoxia did not change MTT-levels *in vitro* (*p* > 0.05).

*In vivo* no significant differences (*p* = 0.96) occurred in MTT-levels after 2 days of Zn administration compared with no Zn intake. Nevertheless, there was a significant correlation between MTT (MTT_1_/MTT_0_) and eGFR (eGFR_1_/eGFR_0_) in case of Zn administration (rho = −0.49; 95%-CI: −0.78 to −0.03; *p* = 0.04).

**Conclusions:**

We found that Zn did induce MTTs *in vitro*, whereas hypoxia had no significant impact. In contrast, no significant increase of MTTs was detected after *in vivo* administration of Zn. However, there was a significant negative correlation between MTT and eGFR *in vivo* in case of Zn administration, this could indicate a protective role of MTTs in a setting of reduced kidney function, which is possibly influenced by Zn.

**Trial registration:**

ClinicalTrials.gov Identifier: NCT00399256. Retrospectively registered 11/13/2006.

## Background

Oxidative stress engendered by hypoxia and inflammation can lead to DNA damage and destruction of cell structures [1, 2]. Metallothioneins (MTTs) are potent endogenous antioxidants that can inactivate the free radicals that mediate oxidative stress [3]. MTTs are a group of 6 –7 kDa polypeptides containing approx. 20 cysteine amino acids [4]. They regulate and control intracellular metal ion metabolism [5] and normally are able to bind zinc in mammals, which mediates the antioxidative protective effect [6]. Beyond that, MTTs potentially play a role in different types of cancer, including breast, prostate, or kidney tumors [7].

MTTs were originally discovered in horse kidneys as a cadmium carrier [8]. Besides many MTT-like proteins, there are 4 different MTTs isoforms discovered so far in humans, MT-1, MT-2, MT-3 and MT-4 with partially different concentrations depending on tissue type [9].

Several groups have reported protective effects of MTTs against oxidative damage and a reduction of oxidative stress [3, 10–12]. Tissue and cells have increased resistance to reactive oxygen species during MTT overexpression [13]. There are several known inducers of MTT polypeptides, including acute exercise in humans [14]. In rats, increased MTT expression occurs during ischemic acute kidney injury [15]. Zinc (Zn) is a strong inducer of MTTs *in vitro* in renal proximal tubular cells [16]. *In vivo,* Sullivan et al. [17] demonstrated that Zn supplementation in healthy participants increased MTT concentration in erythrocytes and monocytes.

MTTs seem to play a major protective role in renal tissues [18–20], and our goal was to further investigate the behavior of MTT under the influence of Zn and hypoxia *in vitro* in renal cells and *in vivo* in serum specimens.

## Methods

### Cell culture

Epithelial renal tubular cells from swine (S*us scrofa*; LLC-PK1) were obtained from A.H. Schinkel (Amsterdam, The Netherlands). The renal cells were cultured in Medium 199 (Invitrogen, Darmstadt, Germany) with added 100 U/ml penicillin, 100 U/ml streptomycin, and 10% fetal bovine serum albumen (Invitrogen, Darmstadt, Germany).

Cultures were incubated at 37 °C in different environments:

I. : 4.5 h under normal conditions in cell culture cabinet (5% CO_2_, 18% O_2_)
II. : 24 h under normal conditions in cell culture cabinet (5% CO_2_, 18% O_2_)
III. : 4.5 h under hypoxia in cell culture cabinet (5% CO_2_, 3% O_2_)
IV. : 24 h under hypoxia in cell culture cabinet (5% CO_2_, 3% O_2_)
V. : 4.5 h under hypoxia (5% CO_2_, 3% O_2_) and 19.5 h under normal conditions in cell culture cabinet (5% CO_2_, 18% O_2_).

Cell cultures were placed in five 6-well plates (Greiner, Darmstadt, Germany), with 1 × 10^5^ cells distributed per well. The swine cells were cultured for 3 days until they were densely populated (up to 70% in each well). The cells were treated with increasing concentrations of zinc sulfate (Merck, Darmstadt, Germany) levels. Specifically, 4 groups of tubular cell cultures were studied, using a control level of 0, and then 5, 10, and 15 μg/ml ZnSO_4_ to quantitate the effects of progressively increasing concentrations of the Zn ion.

### Real-time PCR

RNA was isolated using the RNAeasy mini-kit (Qiagen, Hilden, Germany) according to the manufacturer’s instructions. Only samples with an optical density (OD) of 260/280-ratio between 2.0 and 2.1 were used. RNA quality was analyzed using a Bioanalyzer (Agilent Technologies) and RNA integrity numbers (RIN) > 8 were observed for all RNA samples. Concentrations were ascertained by OD (A_260_ = 1 = 40 μg/ml). For cDNA generation measurements, TaqMan Reverse Transcription Reagents (Applied Biosystems, Foster City, CA, USA) were used. Quantification of gene expression was performed through real-time PCR as previously described [16]. In brief, primers used for real-time PCR were MTT Reverse-Primer: 5′-ATG GAT CCC AAC TGC TCC T-3′; and MTT Reverse-Primer: 5′-CAG CAG CTG CAC TTG TCC-3′. As a control, histone 3.3 was applied: Forward primer: 5′ CCA CTG AAC TTC TGA TTC GC 3′ and histone 3.3 reverse primer: 5′ GCG TGC TAG CTG GAT GTC TT 3′ [16]. Real-time PCR using SYBR green was performed on a 7900 Real-Time PCR System (Applied Biosystems, Foster City, CA, USA) according to manufacturer’s protocol. MTT mRNA levels were normalized to histone 3.3 expression.

### Immunohistochemistry

For IHC analysis, cells were cultivated as described above. In this analysis the environments II, IV and III were used. After 24 h, the cell cultures were prepared for IHC analysis: Cells were trypsinized and washed with culture medium. Cytospin preparation was performed with a Cytofuge 3 (Shandon, USA). Thereafter, samples were dried for 2 h at room temperature and stored at −21 °C.

For analysis, specimens were thawed and fixed with 4% paraformaldehyde. For endogenous peroxidase blocking, 3% H_2_O_2_ in methanol was used. Endogenous biotin was blocked by means of Avidin-Biotin-Blocking Kit (Linaris, USA), according to the manufacturer’s recommendations. Specimen staining was performed by using the ABC method as described previously [21].

### Quantitative staining measurement of IHC MTT expression

MTT immune-histochemical staining was quantified by light microscope (Carl Zeiss AG, Oberkochen, Germany) with an image analysis workstation, a microscope with computerized analysis [22]. For automated evaluation of MTT protein expression, the software-based image analysis system Tissue Studio v.3.6 (Definiens AG, Munich, Germany) was utilized.

The predefined analysis solution “Nuclei, Membranes and Cells” with the tasks of ROI detection, nucleus and membrane detection, and cell classification was used. Threshold settings for nucleus and cytoplasm detection were adjusted. Detected cells were sub-classified as negative, low, medium or high according to IHC staining intensity. The Tissue Studio Score was derived from the number of cells (IHC positive and negative) multiplied by the average IHC marker intensity.

### Study design and subjects

The *in vivo* study is a secondary analysis of a prospective, randomized, double-blind trial on prevention of contrast media-induced nephropathy. In this study 18 patients received 60 mg Zn p.o. QD for 2 days before serum MTT measurements. Previously comparable doses were used [17, 23]. Furthermore we adhered to the lowest observed adverse effect level (LOAEL) of 60 mg/day [24]. At higher oral doses increase of side effects could occur [25]. Patients receiving NAC or placebo (*n* = 36) served as control group. Inclusion criteria were age ≥ 18 years and serum Creatinine (sCr) ≥ 1.2 mg/dl or creatinine clearance < 50 ml/min (measured by a 12- or 24-h urine collection). Exclusion criteria were acute inflammatory disease, use of NSAID or metformin medication up to 3 days before the study, and abnormal findings in physical examination (e.g., signs of dehydration or inflammation). The detailed study protocol and population was as described by Kimmel et al. [26]. Written informed consent was obtained from all patients. The local ethics committee approved the study (Ethics committee of the University of Tuebingen: 296/2003) and it was registered at ClinicalTrials.gov (NCT00399256).

### Laboratory measurements

In the patients, human MTT was measured by ELISA in serum specimens. A commercial kit (USCN Life Science Inc., Wuhan, and Houston, TX, USA) was used for this measurement and was applied according to the instruction manual (Cat. No.: E1119Hu). Zn was analyzed by atomic absorption spectrometry. Estimated glomerular filtration rate (eGFR) was calculated using the Chronic Kidney Disease Epidemiology Collaboration (CKD-EPI) equation [27].

### Statistical analysis

Statistical analysis was performed by using Prism, GraphPad (La Jolla, CA, USA) and R Version 3.3 [28]. To compare means of groups, Welch’s *t*-test and Welch’s one-way ANOVA or Wilcoxon rank-sum test and Kruskal-Wallis test were used. To compare different statistical models with continuous outcomes, including interaction terms and multiple input variables, partial F-tests were performed. To examine variance homogeneity between groups, Bartlett’s and Levene’s tests were applied. To check for normality of distributions, the Shapiro-Wilk test and Q-Q plots were conducted. In cases of non-normally distributed data, log-transformation was considered. For *in vivo* analysis, we considered the ratio of MTT to baseline MTT (MTT_1_/MTT_0_), and the ratio of eGFR (eGFR_1_/eGFR_0_), correspondingly. To obtain well-fitting linear statistical models, these ratios were logarithmized.

## Results

### *In vitro*

#### Metallothionein induction in LLC-PK1 cell culture

##### Immunohistochemical analysis with LLC-PK1

**Fig. 1:**
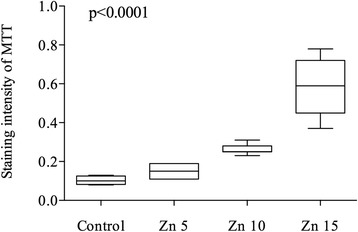
Quantitative Immunohistochemistry Expression of MTT dependent on different Zinc concentrations. Zn: Zinc. Zn 5: 5 μg/ml (17.4 μM); Zn 10: 10 μg/ml (34.8 μM); Zn 15: 15 μg/ml (52.2 μM). Box and whiskers show the interquartile range and total observed range, respectively. Horizontal line within the box shows the median

**Fig. 2:**
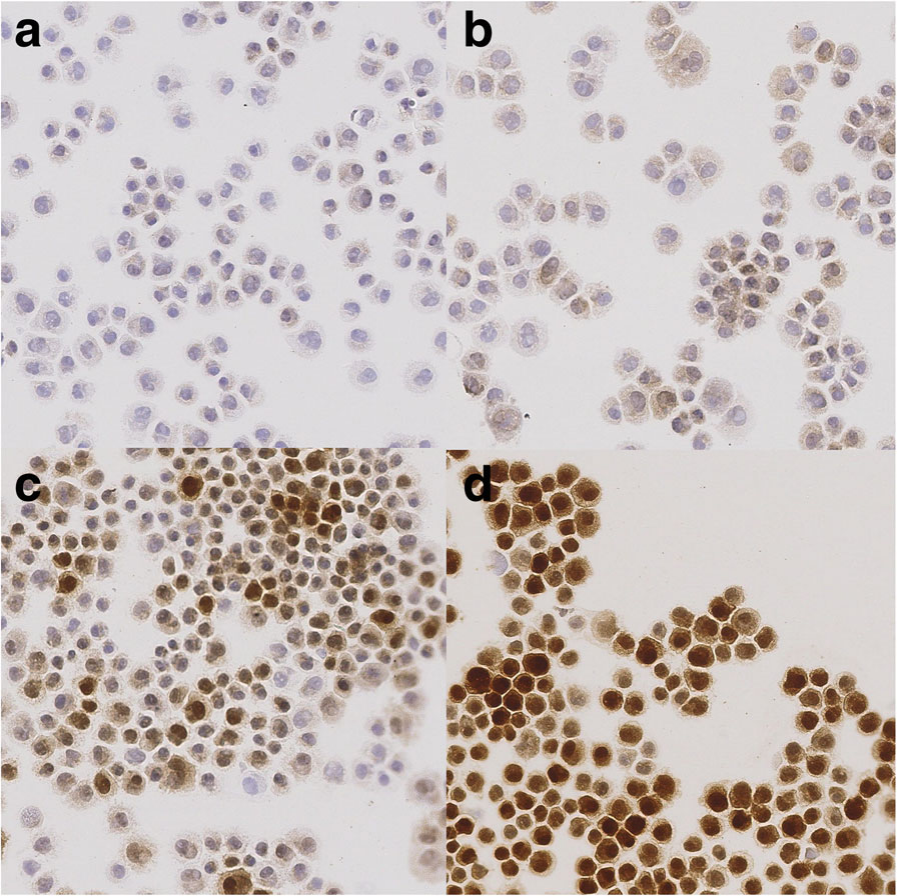
**a**-**d** Quantitative Immunohistochemistry Expression of MTT dependent on different Zinc concentrations. A: control; B: Zinc 5 μg/ml (17.4 μM); C: Zinc 10 μg/ml (34.8 μM); D: Zinc 15 μg/ml (52.2 μM))

**Fig. 3:**
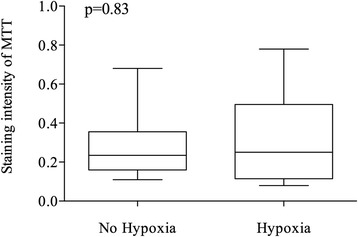
Quantitative Immunohistochemistry Expression of MTT in the presence and absence of hypoxia. Box and whiskers show the interquartile range and total observed range, respectively. Horizontal line within the box shows the median

Quantitative analysis by means of Tissue Studio v.3.6 (Definiens AG, Munich, Germany) showed increasing MTT-expressions with rising Zn concentrations (Fig. 1, Fig. 2, and Table 1). Median (IQR) expression was 0.10 (0.07–0.12) in control, 0.15 (0.12–0.18) in Zn 5 μg/ml (17.4 μM), 0.25 (0.25–0.27) in Zn 10 μg/ml (34.8 μM) and 0.59 (0.48–0.70) in Zn 15 μg/ml (52.2 μM). This increase was statistically significant (*p* < 0.0001). The median quantitative MTT staining intensity of the cell culture cells under hypoxic conditions (IV and V) versus those under a non-hypoxic environment did not differ significantly (*p* = 0.83) (Fig. 3 and Table 2).

**Table 1:** Quantitative Immunohistochemistry Expression of MTT dependent on different Zinc concentrations

Zinc concentration	n	Mean	95% CI	Median	IQR	*p*-value
Control	4	0.10	0.07–0.14	0.10	0.09–0.12	<0.0001
5 μg/ml (17.4 μM)	6	0.15	0.11–0.19	0.15	0.12–0.18	
10 μg/ml (34.8 μM)	7	0.26	0.24–0.28	0.25	0.25–0.27	
15 μg/ml (52.2 μM)	7	0.59	0.45–0.72	0.59	0.48–0.7	

*Zn* Zinc

**Table 2:** Quantitative Immunohistochemistry Expression of MTT dependent on different Zinc concentrations

Hypoxia	n	Mean	95% CI	Median	IQR	*p*-value
no	8	0.285	0.13–0.44	0.235	0.18–0.33	0.83
yes	16	0.309	0.19–0.43	0.25	0.13–0.47	

##### Real-time PCR analysis using LLC-PK1 cells

**Fig. 4:**
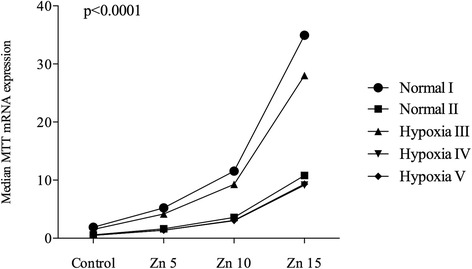
Median mRNA expression of MTT dependent of different Zinc concentrations - Zn 5: 5 μg/ml (17.4 μM); Zn 10: 10 μg/ml (34.8 μM); Zn 15: 15 μg/ml (52.2 μM). Zn: Zinc. Cell culture environments: I: 4.5 h normal conditions in cell culture cabinet (5% CO_2_, 18% O_2_, 37 °C); II: 24 h normal conditions in cell culture cabinet (5% CO_2_, 18% O_2_, 37 °C); III: 4.5 h hypoxia cell culture cabinet (5% CO_2_, 3% O_2_, 37 °C); IV: 24 h hypoxia cell culture cabinet (5% CO_2_, 3% O_2_, 37 °C); V: 4.5 h hypoxia cell culture cabinet (5% CO_2_, 3% O_2_, 37 °C) and 19.5 h normal conditions in cell culture cabinet (5% CO_2_, 18% O_2_, 37 °C)

In real-time PCR analysis, a significant increase of MTT expression was detectable (*p* < 0.0001) under rising ZnSO_4_ concentrations: control; 5 μg/ml (17.4 μM); 10 μg/ml (34.8 μM); and 15 μg/ml (52.2 μM). Median MTT (95% CI) expression for normal environment (I) were 1.90 (1.49–2.43), 5.22 (4.09–6.66), 11.56 (9.06–14.76), and 34.94 (26.85–45.47). For the normal environment with longer incubation (II), median MTT expressions were 0.59 (0.46–0.75), 1.62 (1.27–2.05), 3.58 (2.82–4.55), and 10.81 (8.44–13.85). The three hypoxic environments showed a similar behavior: Under short hypoxic incubation (III), median MTT expression readings were 1.53 (1.20–1.95), 4.18 (3.28–5.34), 9.27 (7.26–11.83), and 28.00 (21.52–36.44), respectively. At longer hypoxic incubation (IV) median MTT expression readings were 0.50 (0.39–0.63), 1.37 (1.08–1.74), 3.03 (2.38–3.85), and 9.15 (7.15–11.72). Hypoxic environment V also showed an increase of median MTT expressions with rising Zn concentrations: 0.51 (0.40–0.65), 1.39 (1.09–1.78), 3.08 (2.42–3.93), and 9.32 (7.16–12.12). The increase was statistically significant (*p* < 0.0001) (Fig. 4 and Table 3).

**Table 3:** Median mRNA expression of MTT dependent on different Zinc concentrations under different hypoxic (III, IV) and non-hypoxic (I, II) environments. Cell culture environments: I: 4.5 h normal conditions in cell culture cabinet (5% CO_2_, 18% O_2_, 37 °C); II: 24 h normal conditions in cell culture cabinet (5% CO_2_, 18% O_2_, 37 °C); III: 4.5 h hypoxia cell culture cabinet (5% CO_2_, 3% O_2_, 37 °C); IV: 24 h hypoxia cell culture cabinet (5% CO_2_, 3% O_2_, 37 °C); V: 4.5 h hypoxia cell culture cabinet (5% CO_2_, 3% O_2_, 37 °C) and 19.5 h normal conditions in cell culture cabinet (5% CO_2_, 18% O_2_, 37 °C)

	Median MTT expression (95% CI)					
	Zinc concentration	Control	5 μg/ml (17.4 μM)	10 μg/ml (34.8 μM)	15 μg/ml (52.2 μM)	*p*-value
Environment	Normal I	1.90 (1.49–2.43)	5.22 (4.09–6.66)	11.56 (9.06–14.76)	34.94 (26.85–45.47)	0.089
	Hypoxia III	1.53 (1.20–1.95)	4.18 (3.28–5.34)	9.27 (7.26–11.83)	28.00 (21.52–36.44)	
	Normal II	0.59 (0.46–0.75)	1.62 (1.27–2.05)	3.58 (2.82–4.55)	10.81 (8.44–13.85)	0.23
	Hypoxia IV	0.50 (0.39–0.63)	1.37 (1.08–1.74)	3.03 (2.38–3.85)	9.15 (7.15–11.72)	
	Normal II	0.59 (0.46–0.75)	1.62 (1.27–2.05)	3.58 (2.82–4.55)	10.81 (8.44–13.85)	0.25
	Hypoxia V	0.51 (0.40–0.65)	1.39 (1.09–1.78)	3.08 (2.42–3.93)	9.32 (7.16–12.12)	

**Fig. 5:**
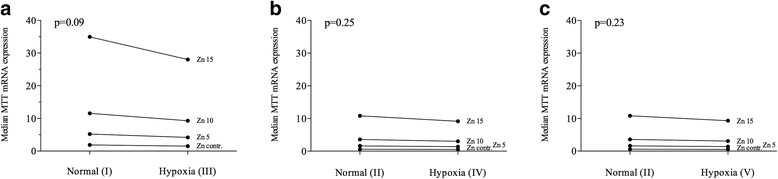
**a**-**c** Median mRNA expression of MTT: The effect of different hypoxic environments (III, IV) compared to non-hypoxic conditions (I, II) with different Zn-concentrations. Zn: Zinc. Zn 5: 5 μg/ml (17.4 μM); Zn 10: 10 μg/ml (34.8 μM); Zn 15: 15 μg/ml (52.2 μM). Cell culture environments: I: 4.5 h normal conditions in cell culture cabinet (5% CO_2_, 18% O_2_, 37 °C); II: 24 h normal conditions in cell culture cabinet (5% CO_2_, 18% O_2_, 37 °C); III: 4.5 h hypoxia cell culture cabinet (5% CO_2_, 3% O_2_, 37 °C); IV: 24 h hypoxia cell culture cabinet (5% CO_2_, 3% O_2_, 37 °C); V: 4.5 h hypoxia cell culture cabinet (5% CO_2_, 3% O_2_, 37 °C) and 19.5 h normal conditions in cell culture cabinet (5% CO_2_, 18% O_2_, 37 °C)

MTT expressions did not differ significantly between hypoxic and non-hypoxic environments. Between Environment I (normal) and III (hypoxic), only a trend to a decreased expression under hypoxic conditions was remarkable (*p* = 0.089). Between II (normal) and IV (hypoxic), a *p*-value of 0.23 was noted between environment II (normal) and IV (hypoxic) and a *p*-value of 0.25 between environment II and V (Table 3 and Fig. 5a, b and c).

### *In vivo*

#### Metallothionein induction in patients

**Fig. 6:**
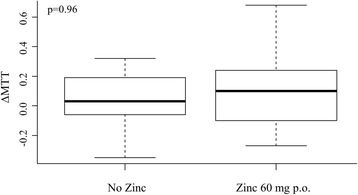
Median ∆MTT in correlation of zinc administration in humans. MTT units: ng/mL; Zn = 0: *n* = 36; Zn = 1: *n* = 18. Box and whiskers show the interquartile range and total observed range, respectively. Horizontal line within the box shows the median

We compared MTT serum levels based on ELISA measurements in patients with moderately impaired kidney function that were receiving low osmolar contrast medium. We measured the increase of MTT (∆MTT) in patients, comparing results between those who received 2 days of treatment with Zn, 60 mg p.o. QD, to those with no Zn supplementation. No significant differences occurred between the two groups (*p* > 0.05) (Fig. 6).

**Fig. 7:**
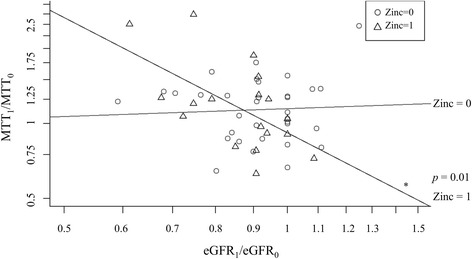
Interaction of MTT (MTT_1_/MTT_0_) and eGFR (eGFR_1_/eGFR_0_) in the presence (∆) and absence (o) of zinc. Correlation of MTT (MTT_1_/MTT_0_) and eGFR (eGFR_1_/eGFR_0_) in case of Zn administration: Rho = −0.49; 95%CI: −0.78 to −0.03; *p* = 0.04. Addition of Zinc has a significant impact (*p* = 0.01). No significant correlation without Zn administration: Rho = −0.01, 95%CI: −0.35 to 0.33; *p* = 0.96. MTT: Metallothionein. eGFR: estimated glomerular filtration rate. Units: MMT: ng/mL; eGFR: mL/min. Zn = 0: *n* = 36; Zn = 1: *n* = 18

We observed a significant negative correlation between MTT (MTT_1_/MTT_0_) and eGFR (eGFR_1_/eGFR_0_) (rho = −0.49; 95%CI: −0.78 to −0.03; *p* = 0.04) in case of Zn administration, Zn had a significant impact on this correlation (*p* = 0.01) (Fig. 7). In absence of Zn, no significant correlation between MTT (MTT_1_/MTT_0_) and eGFR (eGFR_1_/eGFR_0_) could be detected (rho = −0.01, 95%CI: −0.35 to 0.33; *p* = 0.96). Correspondingly, Zn influenced the correlation between MTT (MTT_1_/MTT_0_) and sCr (sCr_1_/sCr_0_) significantly (*p* = 0.01).

Serum Zn levels were measured at baseline and after Zn intake. Median (IQR) increase was +11.5 μg/dl (−7 to 21) in the group with Zn treatment and +3.0 μg/dl (−26 to 22) in those without Zn supplementation. However, this difference was not statistically significant (*p* > 0.05). According to baseline measurements 5/54 (7.4%) subjects had serum Zn levels below reference range (<70 μg/dL).

## Discussion

MTT is an endogenous antioxidant and is known for its protective role against reactive oxygen species [3, 6, 11]. Especially in the kidneys, MTT compounds play a major role, and seem to be involved in renal ageing [18]. Increased expression of MTT occurs in aged kidney tissues in the absence of chronic kidney disease. Predominant expression was described in proximal tubule cells [18]. In experimental settings, MTT induction in the kidney protects the tissues from oxidative stress [19]. In hypoxia, MTTs may play an important protective role: Wu et al. [29] demonstrated in a mouse model that depletion of MTTs worsened hypoxia-induced renal injury, and an increase in MTT-expression stabilizes hypoxia-inducible factor in the kidney [10]. Moreover, kidneys were less susceptible for hypoxia-induced apoptosis in a setting of overexpression of MT2A [18]. Given the known protective effects and the possibility of MTT-induction through Zn, the influence of Zn on diabetic damages was evaluated. In these studies, Zn treatment protected kidneys from diabetic damage [11, 20].

In our *in-vitro* studies, we found that Zn can induce MTT in renal tubular cells, as was reported previously [16]. We found significant increases of MTT levels in quantitative IHC and real-time PCR with augmented Zn concentrations (*p* < 0.0001). In contrast, under hypoxic conditions, no increase of MTT-expression was detected in renal cell cultures by IHC and PCR (*p* > 0.05). This is surprising, because previous studies *in vivo* reported that MTT increased under hypoxic conditions [15].

The interaction between hypoxia and MTT expression may be complex. It is known that many genes are upregulated during hypoxia [10], and overexpression may be mediated by inflammation which results from *in vivo* hypoxia [30]. Inflammation is known to increase MTT levels [31], but inflammation requires the interaction of, among others, inflammatory cells, vessels, and cytokines [32]. *In vitro* this environment does not exist and therefore no increased MTT expression was detectable.

Despite its main intracellular location, MTT also occurred in lower quantity in serum specimens [33, 34]. Similar behaviour of serum MTT in reaction to stress or administered metals suggests that it seems to be adequate to use serum MTT as a marker of intracellular MTT response [35, 36]. Interestingly, when we analyzed blood samples in our prospective study of prevention of contrast media-induced nephropathy, we found that Zn had no measurable effect on MTTs levels in serum samples (*p* > 0.05). This result contradicts previous findings [16, 17]. In summary, according to our present data, induction of MTTs through short-term Zn supplementation does not appear to be significant in patients with impaired kidney function, after exposure to contrast media.

The population and the method of MTT-measurement in the previous *in vivo* studies differed significantly from those in our trial [17]. Whereas Sullivan et al. [17] evaluated healthy participants, our patients presented with pre-existing renal dysfunction. In their study they supplemented over a longer time period and measured MTT in erythrocytes and monocytes. Therefore a comparison seems to be debatable. Additionally, it is possible that the iodinated contrast media could have affected the zinc-MTT-interaction.

Our data indicate that Zn supplementation did not induce MTTs *in vivo* compared with no Zn intake, although we found there was a significant association of MTT with eGFR (rho = −0.49; *p* = 0.04) in case of Zn administration. Zn influenced this correlation significantly (*p* = 0.01). That could imply a negative correlation between eGFR and MTT in reduced kidney function, which could be an indication of a protective role for MTTs. Interestingly, previous studies have shown no MTT increase under chronic hypoxic conditions and during development of chronic kidney disease. Wu et al. [29] demonstrated that during intermittent hypoxia for > 8 weeks accompanied by renal fibrosis, no increase in MTTs was detectable, whereas initially an increased expression of MTTs occurred. Sun et al. [37] also showed that short-term oxidative stress (during hypoxia) induced MTTs, whereas long-term hypoxia did not affect MTTs. The results suggest that in chronic impaired kidney function itself, MTT induction is not evident, whereas in further decline of kidney function, induction of MTTs can be detected in case of Zn administration. We cannot rule out the possibility that a reduced renal excretion could also have influenced serum MTT levels, but there are two observations which do not support these concerns: Zn significantly influences the correlation between eGFR and MTT and in absence of Zn no such association was evident.

We have to emphasize that the increase of Zn in serum did not differ significantly (p > 0.05) between subjects receiving Zn and those who did not. The lacking increase could be originate from the particular issue with Zn serum measurements itself: First, plasma Zn levels are known to change very slowly as response to changes in Zn intake [38] and, after ingestion, Zn is primarily taken up by the liver before a redistribution to the whole body occurs [39]. Second, the plasma Zn amount is less than 1% of the whole Zn storage because it is mainly kept intracellular in the muscles and bones [40] and known to be a poor indicator for total Zn amount. This is due to the fact that the plasma levels are highly affected by multiple factors such as circadian fluctuations and cytokine-related influences [41–43]. Thus, measurements of plasma Zn concentration to assess Zn status are in general known to be diagnostically inconclusive [25].

Nevertheless we cannot exclude that longer or higher doses of Zn supplementation could have led to an increase in plasma Zn which also could have influenced MTT levels. But our study design did not allow for longer intake and the increase of side effects at higher doses did not allow for raising the dosage.

## Conclusions

In conclusion, we found that Zn can induce MTTs *in vitro*, but *in vivo* Zn supplementation of 60 mg per day had no significant effect on MTT-induction. However, there was a significant positive correlation between MTT and eGFR *in vivo* in case of Zn administration, which could indicate a protective role of MTTs in a setting of reduced kidney function, which is possibly influenced by Zn.

## Abbreviations

CKD-EPI: Chronic Kidney Disease Epidemiology Collaboration
IHC: Immunohistochemistry
LOAEL: Lowest observed adverse effect level
MTT: Metallothionein
p.o.: Per os, per mouth
PCR: Polymerase chain reaction
sCr: Serum Creatinine
Zn: Zinc
